# Involvement of GluD2 in Fear-Conditioned Bradycardia in Mice

**DOI:** 10.1371/journal.pone.0166144

**Published:** 2016-11-07

**Authors:** Hiroko Kotajima-Murakami, Sakae Narumi, Michisuke Yuzaki, Dai Yanagihara

**Affiliations:** 1 Department of Life Sciences, Graduate School of Arts and Sciences, The University of Tokyo, Meguro-ku, Tokyo, Japan; 2 Department of Physiology, School of Medicine, Keio University, Shinjuku-ku, Tokyo, Japan; Tokai University, JAPAN

## Abstract

Lesions in the cerebellar vermis abolish acquisition of fear-conditioned bradycardia in animals and human patients. The δ2 glutamate receptor (GluD2) is predominantly expressed in cerebellar Purkinje cells. The mouse mutant *ho15J* carries a spontaneous mutation in GluD2 and these mice show a primary deficiency in parallel fiber-Purkinje cell synapses, multiple innervations of Purkinje cells by climbing fibers, and impairment of long-term depression. In the present study, we used *ho15J* mice to investigate the role of the cerebellum in fear-conditioned bradycardia. We recorded changes in heart rate of *ho15J* mice induced by repeated pairing of an acoustic (conditioned) stimulus (CS) with an aversive (unconditioned) stimulus (US). The mice acquired conditioned bradycardia on Day 1 of the CS-US phase, similarly to wild-type mice. However, the magnitude of the conditioned bradycardia was not stable in the mutant mice, but rather was exaggerated on Days 2–5 of the CS-US phase. We examined the effects of reversibly inactivating the cerebellum by injection of an antagonist against the α-amino-3-hydroxy-5-methyl-4-isoxazole propionate receptor (AMPAR). The antagonist abolished expression of conditioned responses in both wild-type and *ho15J* mice. We conclude that the GluD2 mutation in the *ho15J* mice affects stable retention of the acquired conditioned bradycardia.

## Introduction

The cerebellum plays an important role in motor control and is also involved in the control of cardiovascular functions [1]. The latter role is exemplified by the marked changes in blood pressure and heart rate produced by electrical stimulation of the anterior or posterior cerebellar vermis [2, 3]. The linkage of the cerebellum with heart rate control has been investigated using a classical conditioning paradigm, so-called fear-conditioned bradycardia. Classical conditioning is achieved by pairing a conditioning stimulus (CS) with an unconditioned stimulus (US); this pairing elicits defensive behavioral outcomes such as bradycardia, freezing, and eyeblinking in response to a CS [4]. It has been shown that lesions of the cerebellar vermis severely limit the acquisition and/or expression of conditioned bradycardia in restrained rats and rabbits, without disrupting the baseline heart rate or heart rate responses to the US [5, 6]. Conditioned bradycardia is impaired in patients with medial cerebellar lesions [7]. The Purkinje cells show robust short-latency responses during the CS in conditioned rabbits [8]. In goldfish, cerebellar Purkinje cells show simple spike responses to the CS and complex spike responses to the US during acquisition of fear-conditioned bradycardia [9]. These studies indicate that the cerebellum is involved in fear-conditioned bradycardia. It has been suggested that parallel fibers (PFs) convey the CS and climbing fibers (CFs) transmit the US to the cerebellum during eyeblink conditioning [10]. In an earlier study, we found that a lesion in the inferior olive nuclei of mice disrupted the acquisition and/or expression of conditioned bradycardia and the attenuation of tachycardiac responses to the US [11]. These results raise the question of whether reduction in PF inputs to Purkinje cells and impairment of synaptic plasticity at the PF-Purkinje cell synapses in the cerebellar cortex affect fear-conditioned bradycardia.

The δ2 glutamate receptor (GluD2) is a unique receptor that is selectively expressed in Purkinje cells in the cerebellum and localizes at postsynaptic densities in PF-Purkinje cell synapses, but not at CF-Purkinje cell synapses [12, 13]. GluD2 regulates formation and maintenance of PF-Purkinje cell synapses by binding Cbln1 released from PFs [14]. GluD2^-/-^ mice show approximately 50% fewer PF-Purkinje cell synapses than wild-type mice [15]. In addition, GluD2 is thought to serve as a gatekeeper for long-term depression (LTD) of PF-Purkinje cell synaptic transmission by regulating endocytosis of postsynaptic α-amino-3-hydroxy-5-methyl-4-isoxazole propionate receptors (AMPARs) [16, 17]. GluD2^-/-^ mice also display impaired LTD and ataxic gait [15]. *Hotfoot 4J* (*ho4J*) is a spontaneous mutant in which GluD2 is retained in the endoplasmic reticulum [18]. Interestingly, although *ho4J* mice show normal acquisition of sound-cued fear conditioned responses (freezing), they do not retain the responses at 10 min and 24 h following conditioning [19]. However, it is unclear whether fear-conditioned bradycardia can occur in GluD2 mutants.

*Ho15J* is another spontaneous mutant mouse in which a deletion of exon 2 of the *Grid2* gene results in a 52 amino acid deletion in the leucine/isoleucine/valine binding protein-like domain of the mature protein [20, 21]. Recently, several human families with a similar deletion of the *GRID2* gene have been reported. These patients show ataxia, abnormal eye movements and cognitive delay [22–24]. Thus, in the present study, we aimed to clarify the role of the cerebellum in fear-conditioned bradycardia through use of *ho15J* mice.

## Materials and Methods

### Animals

Twenty-three wild-type and fourteen *ho15J* mice (8–10 weeks of age) were used in the experiments. Animals were housed individually and maintained under a 12 hr light/ 12 hr dark cycle with food and water available ad libitum. The well-being of the mice was carefully monitored, and all efforts were made to minimize the number of animals used and any suffering in the course of the experiments. The present study was approved by the Ethics Committee for Animal Experiments at the University of Tokyo and the Animal Resource Committee of Keio University, and was performed according to the Guidelines for Research with Experimental Animals of the University of Tokyo, the Guidelines set by the Animal Resource Committee of Keio University, and the Guide for the Care and Use of Laboratory Animals (NIH Guide), revised in 1996.

### Surgery

After anesthetizing with 2% isoflurane, mice were restrained in a stereotaxic instrument and the scalp was incised. Chronic electrodes for heart rate recording were anchored with dental acrylic. Fine stainless steel wires were inserted subcutaneously and sewn into the sides of the mice. At the end of surgery, the mice were allowed to recover in a 22°C chamber to avoid hypothermia. The animals were given at least 2 days of recovery before conditioning began.

### Conditioning apparatus

The conditioning apparatus consisted of a restraining device enclosed within a darkened sound-attenuating chamber. The chamber contained two speakers mounted on a two-tier rack. The heart rates of the mice being conditioned were amplified using the implanted chronic electrode connected to an amplifier (MEG-2100; Nihon Kohden, Tokyo, Japan). The output signal from the amplifier was divided into two: one part was monitored on an oscilloscope (VC-6725; Hitachi, Tokyo, Japan) and the other was digitized using an analog to digital converter (MacLab 8s; AD Instruments, Dunedin, New Zealand) and stored on a computer at a 1 kHz sampling frequency. A conditioning (tone) stimulus (CS) and unconditioned (electrical shock) stimulus were delivered using a programmable pulse generator (Master 8; A.M.P.I., Jerusalem, Israel). The tone stimulus was generated by a synthesizer (1941-Wave-Factory; NF Corporation, Yokohama, Japan), amplified by a two-channel power amplifier (SRP-P150; Sony, Tokyo, Japan), and delivered to the mice through two speakers. The sound intensity was measured with a sound-level meter. The unconditioned stimulus was delivered using an electrical stimulator (SEN-2201; Nihon Kohden) connected to two shock electrodes secured around the tail of each mouse.

### Conditioning procedures

Each mouse was habituated to restraint by placing it in a mouse restrainer and attaching electrodes for heart rate recording and tail-shock electrodes; this was carried out twice daily for 60 min. Heart rate was sampled at 90 s intervals throughout each habituation. After habituation, each mouse received 2 consecutive days of unreinforced CS with a fixed 180 s inter-stimulus interval. The CS was a 5 s, 80 dB, 2.5 kHz tone. The conditioning phase was carried out for 5 consecutive days and consisted of 50 trials each day. Each presentation of the CS was followed at offset by a 500 ms, 0.3 mA tail shock US; the trials were performed at 180 s inter-stimulus intervals. The extinction phase was carried out for 5 consecutive days after the conditioning phase, followed by 20 presentations of the CS without the US. In all procedures, except for habituation, the first daily trial was initiated 10 min after the mouse was placed into the chamber without the CS and the US. After the conditioning procedures, the mice underwent a US-alone phase and the tail-flick test to confirm their response to pain stimuli. The US-alone phase consisted of 20 trials in a single day in which only the US was presented. In the tail-flick test, mice received two levels of thermal radiation (80 and 110°C) to the tail and their response latencies were measured.

### Data analysis

Changes in heart rate were assessed by measuring the interbeat interval, defined as the time between successive R-waves (R–R interval) of the cardiac cycle. R-waves of the cardiac cycle were analyzed using LabChart Software (v.3.6.1/s; AD Instruments). The topography of the response to the CS was determined by comparing the mean pre-CS heart rate with each 1 s interval during the 5 s tone. The topography of the response to the US was determined by comparing the mean pre-US heart rate with each 1 s interval of the 6 s following the offset of the US. The occurrence of conditioned bradycardia was defined as a heart rate response during the fifth second of CS that was lower by 1 standard deviation than the mean heart rate responses for each of the 10 trials.

### Injection of an antagonist of AMPAR into the cerebellar cortex

We investigated whether reversible blocking of excitatory synaptic transmissions in the cerebellar cortex contributed to the expression of fear-conditioned bradycardia in *ho15J* mice during the CS-US phase. To this end, we injected NBQX (2,3-dioxo-6-nitro-1,2,3,4-tetrahydrobenzo[f]quinoxaline-7-sulfonamide) into the cerebellar vermis on Day 3 of the CS-US phase in wild-type and *ho15J* mice. Seven wild-type mice (termed wild-type (NBQX) mice) and five *ho15J* mice (termed *ho15J* (NBQX) mice) were used. Surgery and conditioning procedures were performed as described above. Under anesthesia (1.5% isoflurane), 5 μl NBQX sodium solution (100 μM) containing 0.5% pontamine sky blue were injected into the subarachnoid space of the cerebellar vermis (lobules IV-V) at a rate of 1 μl/min using a 33-gauge micro-syringe needle. Six wild-type mice (termed wild-type (saline) mice) received 5 μl of saline/ 0.5% pontamine sky blue delivered in the same manner as in the treated animals. The fear-conditioning experiment was initiated within 30 min after the injection.

### Rotarod

Rotarod tests were performed to measure the effects of NBQX injection. Mice were subjected to a rotarod test on Day 2 of the CS-alone phase and on Days 2, 3, and 4 of the CS-US phase. The rotarod test on each day was conducted 10 min after conditioning. The mice were placed on a 5 cm (diameter) rod that was rotated at 8 rpm. Maximum retention time was set as 120 s. Each mouse was used for 10 rotarod trials except on Day 2 of the CS-US phase, when each mouse was used in 5 trials. Prior to the rotated trials, the mice were examined on the static rod for up to 120 s.

### Histology

After injection of 5 μl 0.5% pontamine sky blue into the cerebellar cortex (lobule IV-V), the mice were intra-cardially perfused with saline, followed by 4% paraformaldehyde in 0.1 M phosphate buffer. The brains were removed and stored in 4% paraformaldehyde in 0.1 M phosphate buffer for 1 day. They were then washed sequentially in 5, 10 and 20% sucrose in phosphate buffer for 7 days at each concentration. Sagittal sections (80 μm-thickness) were then cut from each brain using a cryostat. Sections were cut through the entire injection site and stained with Neutral Red. The sections were then mounted on glass microscope slides and covered with a coverglass. The extent of diffusion of the pontamine sky blue from the injection site was measured under a microscope.

### Statistical Analyses

All data were analyzed using the Statistical Package for Social Sciences (SPSS, Japan, Inc., Tokyo, Japan) version 14.0 using Student’s *t*-test, one-way ANOVA, two-way ANOVA, and three-way ANOVA for repeated measures and the Bonferroni post hoc test. Results are presented as means ± standard error of the mean (SEM) and statistically significant differences are defined as *p* < 0.05.

## Results

### Heart rate responses in the sound-attenuating chamber and to the CS

**Fig 1:**
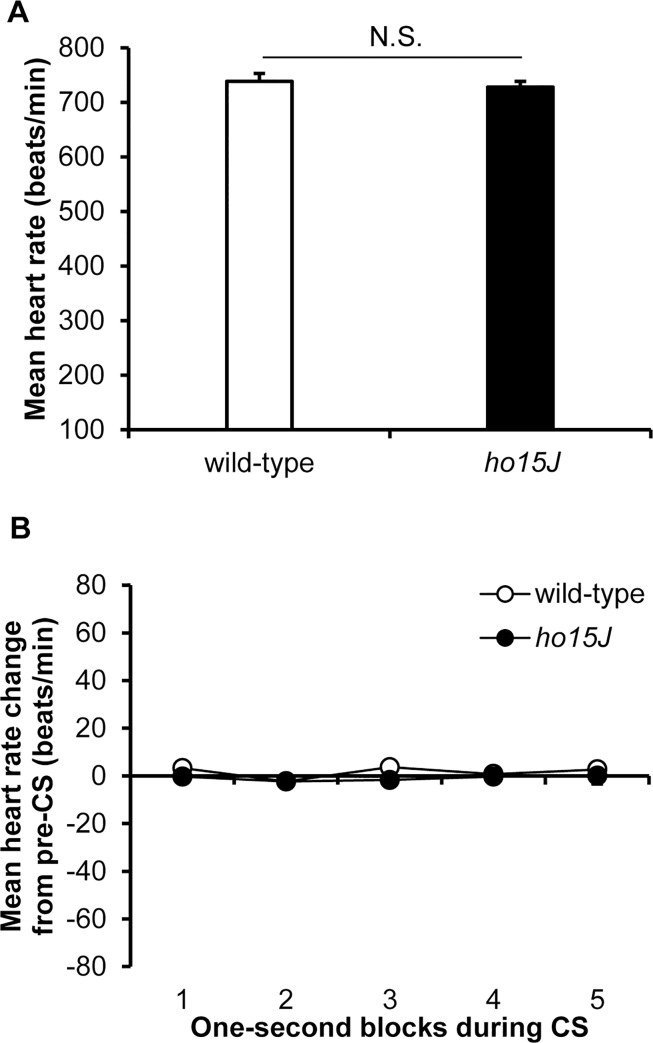
Heart rate responses to the conditioning chamber and to the tone stimulus. (A) Bars show the mean heart rate (n = 50 trials) on the second day of habituation for the wild-type (open bar) and *ho15J* mice (filled bar). (B) Mean change in heart rate (beats/min) during each 1 s period of the 5 s CS collapsed across 50 trials on the second day of CS-alone testing in wild-type (open circles) and *ho15J* mice (filled circles). The horizontal line indicates the final mean heart rate before the 5 s CS collapsed across 50 trials.

Ten wild-type and nine *ho15J* mice were used in the experiments. Wild-type and *ho15J* mice showed similar and consistent patterns of baseline heart rates on the second day of habituation (Fig 1A). The mean heart rate of wild-type mice did not differ significantly from that of *ho15J* mice [wild-type, 738 ± 14 beats/min; *ho15J*, 727 ± 10 beats/min; *t* (17) = 0.565, *p* = 0.578 by two-tailed *t*-test]. On the second day of CS-alone testing, the topography of heart rate responses was examined at 1 s intervals during the 5 s CS in 50 trials. The aggregate patterns of heart rate responses for the two mouse groups are shown in Fig 1B. Both wild-type and *ho15J* mice showed similar heart rate responses [*F*(4, 68) = 1.015, *p* = 0.406 by two-way repeated measure ANOVA] indicating that the *ho15J* mutant mice had stable heart rate responses to the sound-attenuating chamber and the tone-conditioning stimulus.

### Conditioned bradycardia in wild-type and *ho15J* mice during CS-US phase

**Fig 2:**
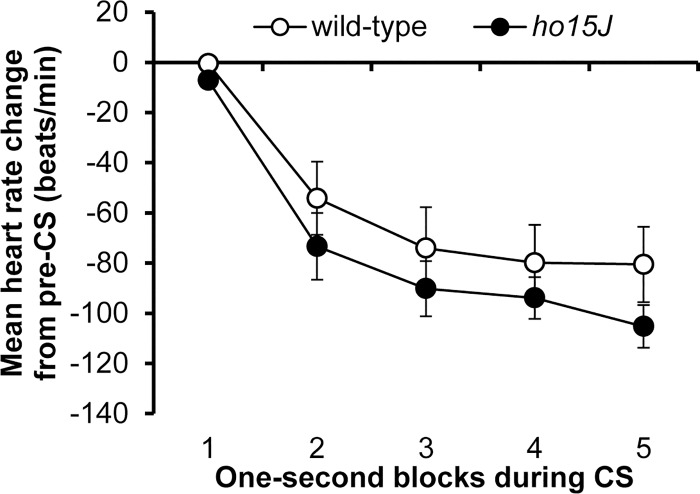
The acquisition of conditioned bradycardia on Day 1 of the CS-US phase. Shown is the mean heart rate change from the pre-CS baseline through the 5 s CS collapsed across 50 trials on Day 1 of the CS-US phase. The wild-type (open circles) and *ho15J* mice (filled circles) demonstrated progressively increasing bradycardia throughout the CS period.

**Fig 3:**
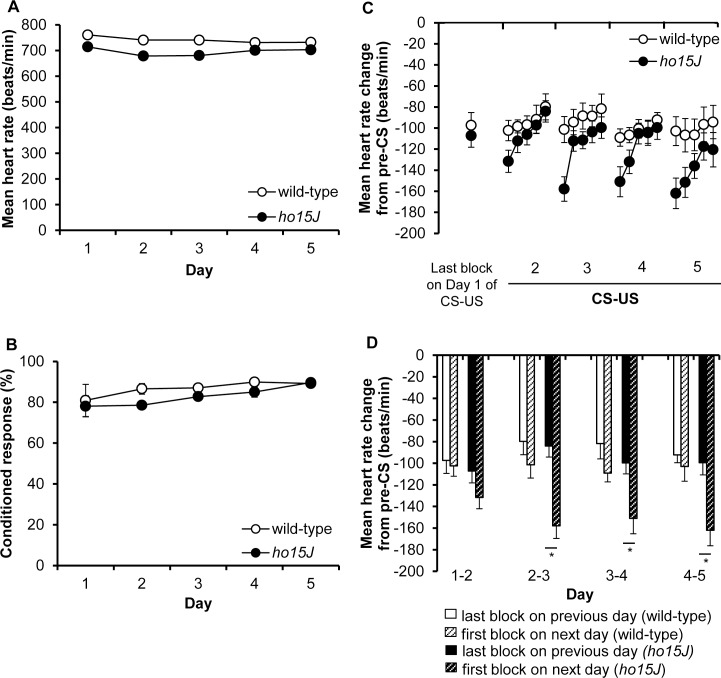
Conditioned bradycardia during the CS-US phase. (A) Mean heart rate for the wild-type and *ho15J* mice during 5 consecutive days of the CS-US phase. (B) Probabilities of conditioned bradycardia for wild-type and *ho15J* mice during the 5 days of the CS-US phase. (C) The conditioned bradycardia for each second-block on each of the 5 consecutive days of the CS-US phase is shown for the wild type and the *ho15J* mice. The mean heart rate change relative to the pre-CS level is shown. (D) The mean heart rate change of the last 10 trials (trial 41–50) on each day and that of the first 10 trials (trial 1–10) on the following day for each type of mouse during the 5 days of the CS-US phase is indicated by the bars. The mean heart rate change relative to the pre-CS level is shown. *p < 0.05.

Conditioned bradycardia was investigated for five consecutive days of the CS-US phase in wild-type and *ho15J* mice. The topography of heart rate responses during CS on Day 1 of the CS-US phase was compared in wild-type and *ho15J* mice; the aggregate patterns from 50 trials are shown in Fig 2. In both wild-type and *ho15J* mice, the dominant responses were a decrease in heart rate, i.e., bradycardia during the five seconds of the CS. There were no significant differences in the conditioned bradycardia during CS between wild-type and *ho15J* mice [*F*(4, 68) = 0.595, *p* = 0.514 by two-way repeated measure ANOVA]. There were no significant differences between wild-type and *ho15J* mice at the 5th second of CS [wild-type mice, -80 ± 15; *ho15J* mice, -105 ± 8.4; *t* (17) = 0.565, *p* = 0.578 by two-tailed *t*-test]. Baseline heart rates during the 5 days of the CS-US phase did not differ between wild-type and *ho15J* mice [*F*(4, 68) = 1.008, *p* = 0.382 by two-way repeated measure ANOVA; Fig 3A]. The probabilities of occurrence of conditioned bradycardia on each of the five days of the CS-US phase are shown in Fig 3B. Both wild-type and *ho15J* mice showed conditioning with no significant difference between the two groups [*F*(4, 32) = 0.828, *p* = 0.517 by two-way repeated measure ANOVA]. These results indicate that the *ho15J* mice acquired conditioned bradycardia at the same rate as wild-type mice. We compared conditioned bradycardia during Days 2–5 of the CS-US phase after acquisition in the two groups of mice; conditioned bradycardia in each group over the 5 days of the CS-US phase is shown in Fig 3C and 3D. The data points in the Figures represent the means from 10 trials collected at the 5th second of the CS in each trial. On Days 2–5 of the CS-US phase, the *ho15J* mice had significantly different response curves to the wild-type mice [*F*(4, 345) = 3.104, *p* = 0.016 by three-way repeated measure ANOVA; Fig 3C]. In daily trials, conditioned bradycardia in *ho15J* mice occurred at similar levels to that in wild-type mice. Wild-type mice exhibited stable conditioned bradycardia when the last block on one day was compared to the first block on the next [Day 1–2 of CS-US, -97 ± 12, -102 ± 9, *t*(18) = 0.324, *p* = 0.75; Day 2–3 of CS-US, -79 ± 12, -101 ± 12, *t*(18) = 1.237, *p* = 0.232; Day 3–4 of CS-US, -81 ± 14, -109 ± 8, *t*(18) = 1.667, *p* = 0.113; Day 4–5 of CS-US, -92 ± 7, -102 ± 13, *t*(18) = 0.684, *p* = 0.503 by two-tailed *t*-test; Fig 3D]. By contrast, *ho15J* mice showed rather enhanced bradycardia on the first block of each day compared to the last block on the previous day [Day 1–2 of CS-US, -107± 10, -131 ± 10, *t*(16) = 1.604, *p* = 0.128; Day 2–3 of CS-US, -84 ± 10, -141 ± 19, *t*(16) = 2.616, *p* = 0.019; Day 3–4 of CS-US, -99 ± 10, -150 ± 14, *t*(15) = 2.799, *p* = 0.013; Day 4–5 of CS-US, -99 ± 11, -161 ± 14, *t*(16) = 3.411, *p* = 0.04 by two-tailed *t*-test; Fig 3D]. These results indicate that conditioned bradycardia in *ho15J* mice was not retained appropriately from the last block of each day to the first block on the following day.

### Extinction of conditioned bradycardia in wild-type and *ho15J* mice

**Fig 4:**
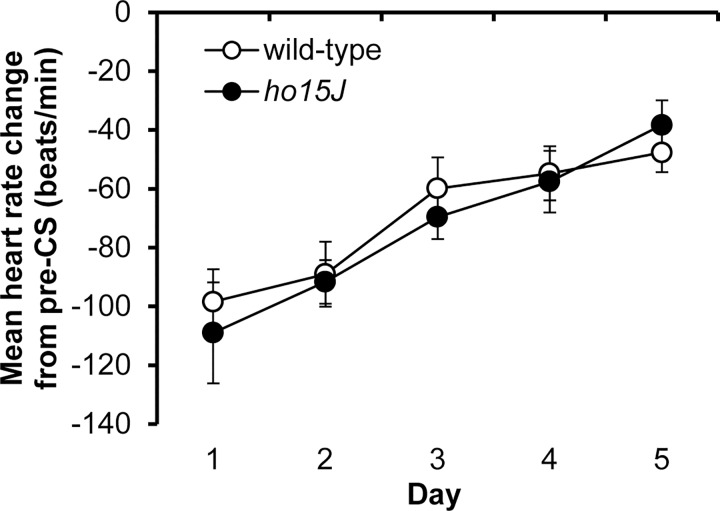
Extinction of conditioned bradycardia. Shown is the daily mean change in heart rate (beats/min) during the 5 s CS collapsed across daily 20 trials during the five days of the extinction phase.

After the CS-US phase, the mice were allowed five consecutive days of extinction. Both wild-type and *ho15J* mice showed gradual extinction of their conditioned bradycardia over this five day period with no significant differences between genotypes [*F*(4, 68) = 2.394, *p* = 0.059 by two-way repeated measure ANOVA; Fig 4]. Thus, the mutation of GluD2 in the *ho15J* mice did not affect the rate of extinction of conditioned bradycardia.

### Response to pain stimuli

**Fig 5:**
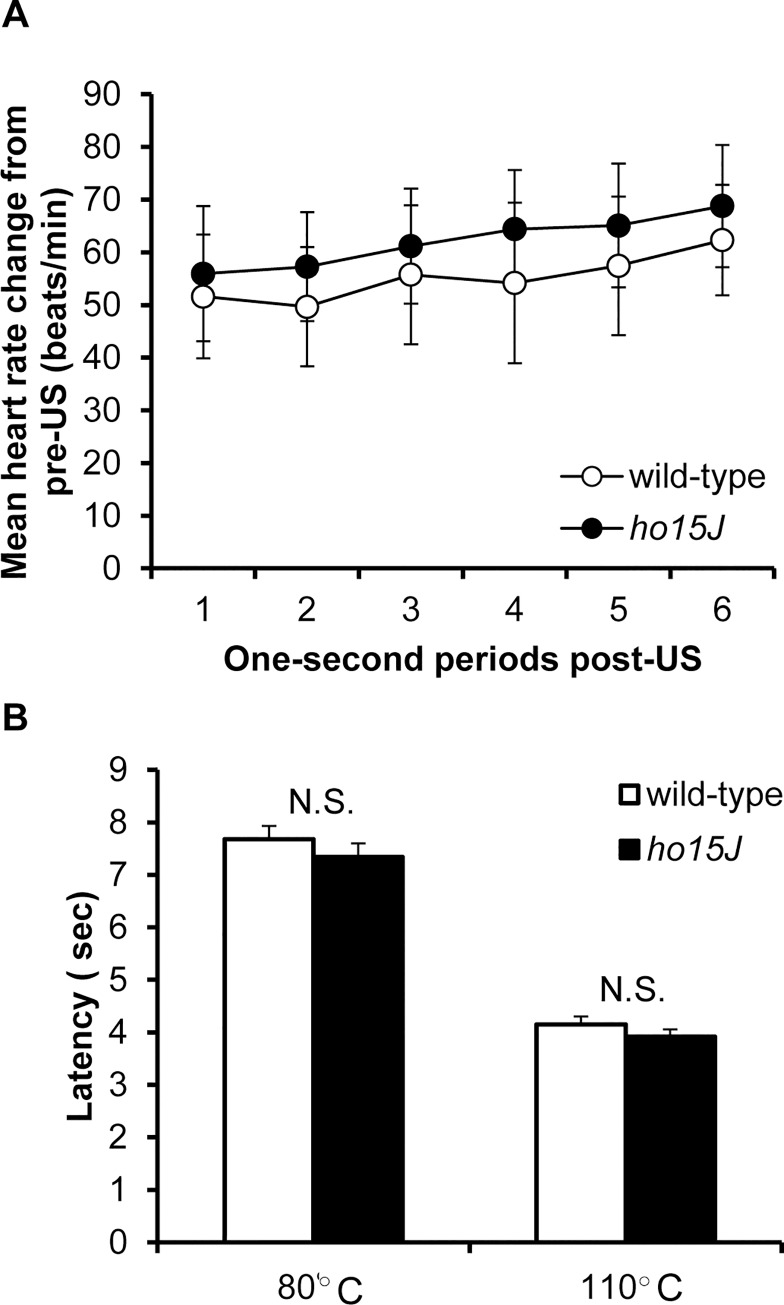
Heart rate responses following a tail shock and responses to pain stimuli. (A) Topography of heart rate responses following application of a tail-shock as the US, during US-alone testing. Mean changes in heart rate (beats/min) during each 1 s period of the 6 s following the offset of the US collapsed across 20 trials. (B) Tail-flick test. Wild-type and *ho15J* mice received 2 levels of thermal stimulus (80°C or 110°C).

After completion of the conditioning procedure, mice were subjected to US-alone and tail flick tests to confirm their responsiveness to pain stimuli. The wild-type and *ho15J* mice showed no differences in tachycardia responses to US-alone [*F*(5, 65) = 0.226, *p* = 0.950 by two-way repeated measure ANOVA; Fig 5A] or to tail-flick tests [80°C: wild-type, 7.6 ± 0.2, *ho15J*, 7.3 ± 0.2, *t*(17) = 0.848, *p* = 0.408 by two-tailed *t*-test, 110°C: wild-type, 4.1 ± 0.1, *ho15J*, 3.9 ± 0.1, *t*(17) = 0.805, *p* = 0.432 by two-tailed *t*-test; Fig 5B]. The *ho15J* mice therefore did not impair heart rate responses to the electrical shock used as the US in fear-conditioning or the responses to a tail shock.

### Effect of NBQX injection into the cerebellum on Day 3 of the CS-US phase

The *ho15J* mice acquired conditioned bradycardia on Day 1 of the CS-US phase and showed abnormal conditioned bradycardia on Days 2–5 of the CS-US phase.

**Fig 6:**
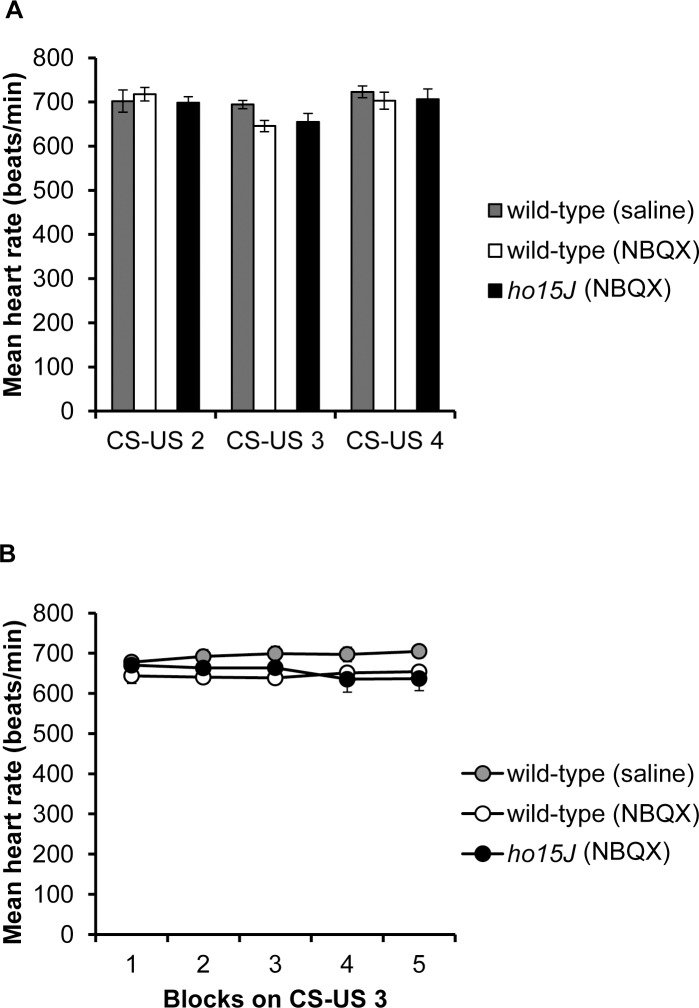
The effects of NBQX injection on mean heart rate. (A) Mean heart rate for the wild-type (saline), wild-type (NBQX) and *ho15J* (NBQX) mice during Days 2–4 of the CS-US phase. NBQX was injected into the wild-type (NBQX) and *ho15J* (NBQX) mice on Day 3 of the CS-US phase. The wild-type (saline) mice were injected with saline. (B) Baseline heart rate for the wild-type (saline), wild-type (NBQX) and *ho15J* (NBQX) mice during Day 3 of the CS-US phase.

**Fig 7:**
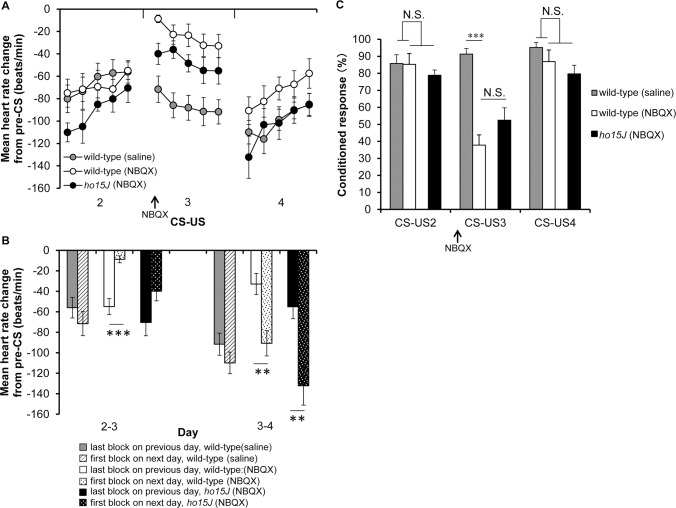
The effects of NBQX injection on Day 3 of the CS-US phase. (A) The conditioned bradycardia for the second-block on each interval at Days 2–4 of the CS-US phase is shown for the wild-type (saline), wild-type (NBQX) and the *ho15J* (NBQX) mice. The mean heart rate change relative to the pre-CS level is shown. (B) The mean heart rate change of the last 10 trials (trial 41–50) on each day and that of the first 10 trials (trial 1–10) on the following day for each type of mouse during Days 2–4 of the CS-US phase is indicated by the bars. The mean heart rate change relative to the pre-CS level is shown. **p < 0.01, ***p < 0.0001. (C) Probability of the occurrence of conditioned bradycardia for the wild-type (saline), wild-type (NBQX) and *ho15J* (NBQX) mice during Days 2–4 of the CS-US phase. ***p < 0.0001.

**Fig 8:**
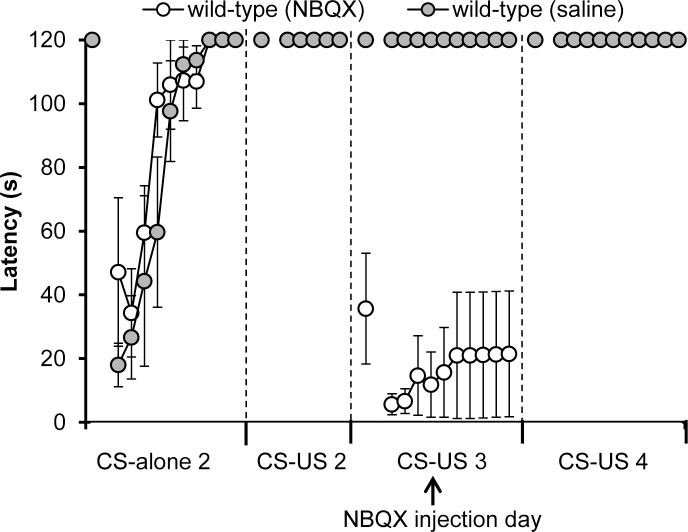
The effects of NBQX injection on performance in a rotarod test. Results of rotarod tests at a speed of 8 rpm for wild-type (NBQX) (open circles) and control wild-type (saline) mice (gray circles). The mice were allowed a maximum retention time of 120 s per trial. The first plotted point in each phase represents data of the static rod.

**Fig 9:**
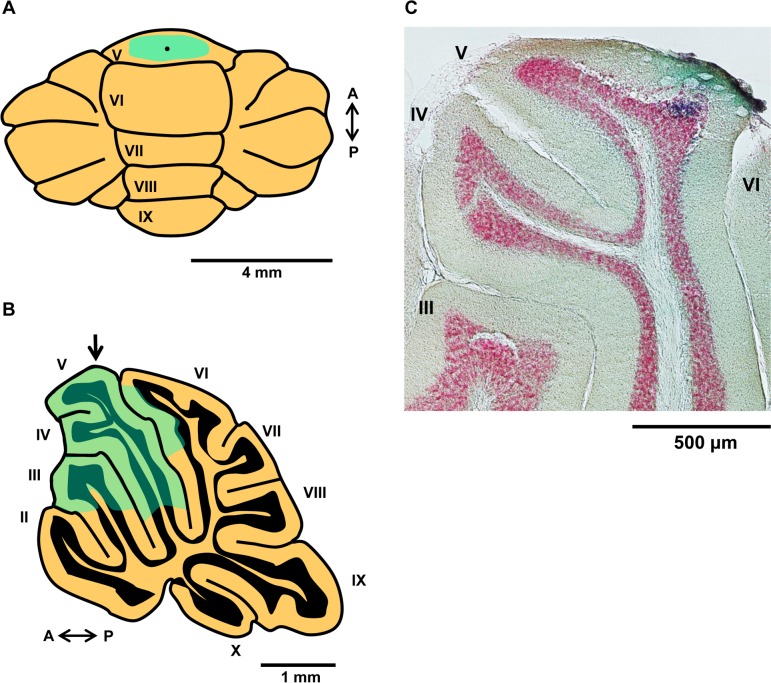
Injection site and diffusion of NBQX in the cerebellum. (A) Schematic showing the injection site and diffusion of pontamine sky blue in a dorsal view of the cerebellum. The black dot in the blue area indicates the injection site and the blue area shows the extent of diffusion of pontamine sky blue. The pontamine sky blue was confirmed to extend from the cerebellar vermis to the intermediate cerebellum. (B) Schematic showing the injection site and diffusion of pontamine sky blue in a sagittal section of the cerebellum. Arrow shows the injection site. Pontamine sky blue was confirmed to extend from cerebellar lobules III to VI. (C) Sagittal section was selected at 0.48 mm from midline. Blue dye is pontamine sky blue and red dye is Neutral Red.

To investigate whether conditioned bradycardia in *ho15J* mice was derived from the cerebellum, we next injected an AMPAR inhibitor NBQX on Day 3 of the CS-US phase; injection of the AMPAR inhibitor into conditioned rabbits temporarily attenuates the expression of conditioned responses in the eyeblink conditioning paradigm [25]. Mean heart rates during Days 2–4 of the CS-US phase did not differ among wild-type (saline), wild-type (NBQX) and *ho15J* (NBQX) mice (see Methods for explanation of the treatment groups) [CS-US 2; *F*(2, 15) = 0.368, *p* = 0.698, CS-US 3; *F*(2, 15) = 2.913, *p* = 0.085, CS-US 4; *F*(2, 15) = 0.532, *p* = 0.598 by one-way ANOVA; Fig 6A]. We analyzed baseline heart rates in each block on Day 3 of the CS-US phase (NBQX injection day) (Fig 6B). The data points represent the means from 10 trials collected at one second prior to the CS in each trial. There were no significant differences among wild-type (saline), wild-type (NBQX) and *ho15J* (NBQX) mice [*F*(2, 15) = 2.781, *p* = 0.094 by two-way repeated measure ANOVA; Fig 6B]. These results suggest that surgery, anesthesia and NBQX injection did not influence the baseline heart rate in wild-type (saline), wild-type (NBQX) and *ho15J* (NBQX) mice. We showed that conditioned bradycardia occurred in each group during Days 2–4 of the CS-US phase (compare Fig 7A and 7B to Fig 3C and 3D). On Day 2 of the CS-US phase (prior to NBQX injection), conditioned bradycardia in *ho15J* (NBQX) mice occurred at similar levels to that in wild-type (saline) and wild-type (NBQX) mice in daily trials, similar to that shown in Fig 3C. However, on Day 3 of the CS-US phase (NBQX injection day), wild-type (NBQX) mice showed attenuated conditioned bradycardia compared to wild-type (saline) mice [*F*(2, 12) = 11.348, *p* = 0.002 by two-way repeated measure ANOVA; Fig 7A]. There were no significant differences in conditioned bradycardia between wild-type (NBQX) and *ho15J* (NBQX) mice [*p* = 0.392 by two-way repeated measure ANOVA; Fig 7A]. On Day 4 of CS-US phase (the Day following NBQX injection), there were no significant differences in conditioned bradycardia among the mice [*F*(2, 13) = 1.367, *p* = 0.289 by two-way repeated measure ANOVA; Fig 7A]. On both Day 2 and Day 4 of the CS-US phase, wild-type (NBQX) mice showed similar conditioned bradycardia responses [*F* = 0.266, *p* = 0.616 by two-way repeated measure ANOVA; Fig 7A]. Similar results were also shown in the *ho15J* (NBQX) mice [*F* = 0.025, *p* = 0.880 by two-way repeated measure ANOVA; Fig 7A]. Wild-type (saline) mice exhibited stable conditioned bradycardia when the last block on one day was compared to the first block on the next [Day 2–3 of CS-US, -56 ± 10, -71 ± 11, *t*(10) = 1.004, *p* = 0.339; Day 3–4 of CS-US, -91 ± 10, -109 ± 10, *t*(10) = 1.207, *p* = 0.255; Fig 7B]. However, wild-type (NBQX) mice showed a reduction in conditioned bradycardia on the first block on Day 3 of the CS-US phase compared to that of the last block on Day 2 of the CS-US phase [*t*(12) = -5.431, *p* < 0.0001; Fig 7B]. There were no significant differences between the last block on Day 2 of the CS-US phase and the first block on Day 3 of the CS-US phase in *ho15J* (NBQX) mice [*t*(6) = -1.941, *p* = 0.100; Fig 7B]. Both wild-type (NBQX) mice and *ho15J* (NBQX) mice showed an increased conditioned bradycardia on the first block on Day 4 of the CS-US phase compared to the last block on Day 3 of the CS-US phase [wild-type (NBQX); *t*(11) = 3.414, *p* = 0.006, *ho15J* (NBQX); *t*(8) = 3.479, *p* = 0.008; Fig 7B]. Fig 7C shows the probability of occurrence of conditioned bradycardia for each type of mouse during Days 2–4 of the CS-US phase. There were no significant differences in the probability of occurrence of conditioned bradycardia between the three groups on Day 2 of the CS-US phase [*F*(2, 15) = 0.447, *p* = 0.647 by one-way ANOVA; Fig 7C]. On Day 3 of the CS-US phase (the NBQX injection day), the wild-type (NBQX) mice showed a reduction in the probability of occurrence of conditioned bradycardia compared to that of the wild-type (saline) mice [*p* < 0.0001 by Bonferroni post-hoc test]. There were no significant differences in the probability of occurrence of conditioned bradycardia between the wild-type (NBQX) mice and the *ho15J* (NBQX) mice [*p* = 0.286 by Bonferroni post-hoc test]. On the following day, there were no significant differences in the probability of occurrence of conditioned bradycardia between the wild-type (saline) and the wild-type (NBQX) mice [*p* = 0.866 by Bonferroni post-hoc test]. The levels of fear-conditioned bradycardia in the wild-type mice (NBQX) were not significantly different from those in the *ho15J* (NBQX) mice [*p* = 0.1000 by Bonferroni post-hoc test]. We confirmed the effect of NBQX injection on locomotor performance of the wild-type (saline) mice and the wild-type (NBQX) mice using a rotarod test. The results are shown in Fig 8, in which the first plotted point in each phase reflects data using the static rod. The wild-type (NBQX) mice showed a worse performance on Day 3 of the CS-US phase than the wild-type (saline) mice [*F* (1, 7) = 6.912, *p* = 0.034 by two-way repeated measure ANOVA, static rod *t*(5) = -3.132, *p* = 0.026 by two-tailed *t*-test, Fig 8], however, the performance of the wild-type (NBQX) mice recovered to a level that was similar to that of the wild-type (saline) mice on the following day. A histological analysis confirmed that pontamine sky blue was present in a diffuse pattern from the cerebellar vermis to the intermediate cerebellum following injection (Fig 9A). In a sagittal section, the pontamine sky blue was present from lobules III to VI (Fig 9B and 9C). These results indicate that conditioned bradycardia in the *ho15J* mice was determined by the cerebellum just as in the wild-type mice.

## Discussion

In the present study, we show that fear-conditioned bradycardia responses could be acquired, expressed and extinguished in *ho15J* mice, a GluD2 mutant mouse similar to certain human patients. Unlike wild-type mice, however, *ho15J* mice showed enhanced bradycardia on the first block of trials during Days 2–5 of CS-US phase. In contrast, in the fear-conditioned freezing, although *ho4J* mice could acquire the conditioned freezing response, they could not retain it 10 min and 24 h after the conditioning [19]. Interestingly, mature wild-type mice which have been injected with an anti-GluD2 antibody (anti-δH2) have a similar ability to remain on a rotarod as control mice; however, 24 h after injection, their performance was poorer than that of control mice [26]. Similarly, immature TgR/K mice, in which D-Ser-dependent LTD is impaired, performed more poorly on the first block every day during 5-day sessions [27]. The results reported in the previous studies are similar to those obtained here, but it remains unclear why fear-conditioned bradycardia is enhanced, rather than impaired, as in the case for fear-conditioned freezing and motor learning using rotarod tests. CCAAT/enhancer binding protein δ (CyEBPd) is a transcriptional regulator and widely expressed in the central nervous systems [28]. Mice with a targeted deletion of the CyEBPd gene show enhanced contextual fear responses 24 hr after training [29], suggesting that enhanced fear responses are produced by deletion of a transcriptional regulator. Although no specific explanation can yet be provided, it is interesting to speculate that signaling pathways involving GluD2 may be required for mice to stably retain the fear-memory.

We showed that fear-conditioned bradycardia was attenuated by injection of NBQX into the cerebellum of wild-type and *ho15J* mice on Day 3 of the CS-US phase. It has been reported that injection of AMPAR inhibitor into a conditioned rabbit temporarily attenuates the expression of conditioned responses in eye-blink conditioning [25]. Here, we found that on Day 4 of the CS-US phase, the *ho15J* mice showed conditioned bradycardia similar to that prior to the NBQX injection day (Day 2 of the CS-US phase). This finding indicated that there were no effects such as irreversible damage of the retention process of fear-conditioned bradycardia in *ho15J* mice during Days 2–4 of the CS-US phase. Thus, we suggest that reversible blocking of AMPAR by injection of NBQX in *ho15J* mice affected the expression of acquired conditioned bradycardia, but did not affect the retention of acquired conditioned bradycardia. The cerebellum has previously been shown to be important for fear-conditioned freezing responses and post-retrieval processes in conditioned rats, while synthesis of proteins in the cerebellum is required for retrieved fear memory [30]. It is believed that the cerebellum may have an important role in the retention of fear memory in fear conditioned bradycardia.

The role of synaptic plasticity (e.g., long-term potentiation (LTP) vs. LTD) at PF-Purkinje cell synapses in cerebellum-dependent learning is contentious. Many genetically engineered mice, in which molecules required for LTD induction are modified, display impaired motor learning, such as eyeblink conditioning and adaption of the optokinetic and vestibulo-ocular reflexes [31]. Recently, GluA2-K882A knock-in mice, in which LTD is abolished, were found to display normal motor learning [32]. Conversely, Purkinje cell-specific calcineurin knockout mice, which have a normal LTD but disrupted LTP, show impaired motor learning [33]. One of the confounding factors is compensation by parallel backup pathways for motor learning in genetically modified mice. However, we showed that fear-conditioned bradycardia was attenuated similarly by the injection of NBQX in the cerebellum in both wild-type and *ho15J* mice. We confirmed that the injected NBQX solution spread from the site of injection into the cerebellum. The effect of NBQX injection was also confirmed by its effect on the motor performance in a rotarod test. These results indicate that fear-conditioned bradycardia during the CS-US phase was caused by intrinsic mechanisms within the cerebellum in *ho15J* as well as in wild-type mice. Because LTD is impaired in *ho15J* mice [20], LTD may not play a crucial role in acquisition of fear-conditioned bradycardia.

Acquisition and extinction of conditioned freezing responses are reported to be mediated by LTP and depotentiation in the lateral amygdala, respectively [34]. Although acquisition of eyeblink conditioning was severely impaired in Cbln1-null mice, in which the Cbln1-GluD2 signaling pathway is disrupted, extinction of learned eyeblink responses occurred normally in these mice [35]. In the current study, we also found that the absence of GluD2 in *ho15J* mice did not affect the extinction of conditioned bradycardia responses, indicating that Cbln1-GluD2 signaling might be dispensable for extinction of conditioned responses in mice.

In eyeblink conditioning, it has been shown that the CS is conveyed to the cerebellum via PFs, and that the CFs relay the US to the cerebellum [10]. Purkinje cells show simple spike responses to the CS and complex spike responses to the US during acquisition of fear-conditioned bradycardia in goldfish [9]. Although PF-Purkinje cell synapses are reduced to about 50% and LTD is impaired at PF-Purkinje cell synapses [20, 21], the heart rate responses of *ho15J* mice were associated with the CS tone stimulus. These results indicate that cerebellar circuits might differentially mediate various forms of learning. Further studies are warranted to better understand physiological and pathological mechanisms by which the cerebellar circuit mediates different aspects of learning and motor coordination.

In the present study, we found that *ho15J* mice showed acquisition of conditioned bradycardia on Day 1 of the CS-US phase and that they showed aberrant retention of conditioned bradycardia during Days 2–5 of the CS-US phase. Conditioned bradycardia was attenuated by the injection of NBQX into the cerebellum of *ho15J* as well as in wild-type mice. Therefore, we conclude that the GluD2 signaling pathway plays a crucial role in stable retention of the acquired conditioned bradycardia.
